# The role of ventral tegmental area in chronic stroke rehabilitation: an exploratory study

**DOI:** 10.3389/fneur.2023.1270783

**Published:** 2023-12-05

**Authors:** Loukas G. Astrakas, Sabrina Elbach, Irini Giannopulu, Shasha Li, Howard Benjafield, A. Aria Tzika

**Affiliations:** ^1^Medical Physics, Faculty of Medicine, University of Ioannina, Ioannina, Greece; ^2^Athinoula A. Martinos Center of Biomedical Imaging, Department of Radiology, Massachusetts General Hospital, Harvard Medical School, Boston, MA, United States; ^3^NMR Surgical Laboratory, Department of Surgery, Massachusetts General Hospital, Harvard Medical School, Boston, MA, United States; ^4^Clinical Research and Technological Innovation, Paris, France; ^5^School of Social Sciences and Professions – Psychology, London Metropolitan University, London, United Kingdom

**Keywords:** chronic stroke, Fugl-Meyer upper extremity scale, psychophysiological interaction, ventral tegmental area, reward, rehabilitation

## Abstract

**Introduction:**

The acknowledged role of external rewards in chronic stroke rehabilitation, offering positive reinforcement and motivation, has significantly contributed to patient engagement and perseverance. However, the exploration of self-reward’s importance in this context remains limited. This study aims to investigate the functional connectivity of the ventral tegmental area (VTA), a key node in the brain’s reward circuitry, during motor task-based rehabilitation and its correlation with the recovery process.

**Methods:**

Twelve right-handed healthy volunteers (4 men, 8 women, aged 57.4 ± 11.3 years) and twelve chronic stroke patients (5 men, 7 women, aged 48.1 ± 11.1 years) with clinically significant right-sided motor impairment (mean FM-UE score of 27.6 ± 8.7) participated. The analysis employed the CONN toolbox to assess the association between motor tasks and VTA connectivity using psychophysiological interaction (PPI).

**Results:**

PPI analysis revealed motor-dependent changes in VTA connectivity, particularly with regions within the motor circuitry, cerebellum, and prefrontal cortex. Notably, stronger connectivity between the ipsilesional VTA and cerebellum was observed in healthy controls compared to chronic stroke patients, highlighting the importance of VTA-cerebellum interactions in motor function. Stroke patients’ motor performance was associated with VTA modulation in areas related to both motor tasks and reward processing, emphasizing the role of self-reward processes in rehabilitation. Changes in VTA influence on motor circuitry were linked to improvements in motor performance resulting from rehabilitation.

**Discussion:**

Our findings underscore the potential of neuroimaging techniques in quantifying and predicting rehabilitation outcomes by examining self-reward processes. The observed associations between VTA connectivity and motor performance in both healthy and stroke-affected individuals emphasize the role of psychological factors, particularly self-reward, in the rehabilitation process. This study contributes valuable insights into the intricate interplay between reward circuits and motor function, highlighting the importance of addressing psychological dimensions in neurorehabilitation strategies.

## 1 Introduction

Stroke is a devastating event, recognized as the second leading cause of mortality worldwide and a major cause of chronic disability. Current evidence supports the hypothesis that long-term post-stroke disabilities can potentially be improved through rehabilitation interventions (1). However, the triage of chronic patients who could benefit from rehabilitation and the customization of rehabilitation programs remain critical medical challenges (2). This is primarily due to the complex nature of stroke recovery, which relies on multiple factors such as genetics, pathophysiology, sociodemographics, and therapeutic interventions. Of particular significance are the mood problems and psychological factors, including anxiety and depression, which are commonly observed in chronic stroke patients and heavily influence rehabilitation outcomes (3). These issues can negatively impact patient adherence and the level of participation in rehabilitation programs, highlighting the importance of addressing them in the overall treatment approach.

Introducing rewards has been proved to be a successful strategy to boost motivation, increase engagement, and improve performance during a motor task (4, 5). Human studies have shown that motor cortex excitability depends on motivation (5) and reward probability (6, 7). These results illustrate that motor cortical physiology integrates cognitive mechanisms related to reward valuation. In neuroanatomical terms, this is supported by the dense innervation of the motor cortex from the ventral tegmental area (VTA), which is one of the principal dopaminergic areas of the brain’s reward system. Many animal studies have demonstrated the role of dopamine in motor learning and in modulating motor responses to reward cues (8–11). Other studies suggest that stroke impairs the dopaminergic pathways, resulting in recovery problems (12). In stroke, levodopa treatment showed promising results in rats (13) but clinically unconvincing outcomes in humans (14). In contrast, endogenous dopamine appears crucial for motor skill recovery after stroke, both for animals (15) and humans performing tasks with reward feedback (4, 16–18).

The role of reward brain areas in neurorehabilitation is not yet fully understood. Specifically, it remains unclear whether the dopaminergic regions respond to a rehabilitation task even without extrinsic reward, and whether they contribute to the recovery potential through intrinsic reward processes. Although functional neuroimaging provides connectivity tools to directly assess the association between motor tasks and connectivity of reward areas, these tools have remained largely unexploited. To bridge this knowledge gap, we employed an analysis of psychophysiological interaction (PPI) to reveal areas that undergo motor-dependent changes in their connectivity with VTA in both chronic stroke patients (CSPs) and healthy age-matched control subjects (HCs). Unlike common resting-state connectivity methods, PPI is more suitable for rehabilitation studies as it allows for the exploration of task-dependent connectivity changes between brain regions (19, 20). Using a rehabilitation protocol based on an MR-compatible robotic device (21), we conducted an exploratory study to test the hypothesis that the functional connectivity of the VTA would be modulated by the motor task during rehabilitation and would be related to the recovery process.

## 2 Materials and methods

### 2.1 Subjects

This study enrolled twelve chronic stroke patients (5 men, 7 women, 48.1 ± 11.1 years old) who were recruited through stroke survivor registries at Massachusetts General Hospital, along with twelve age-matched, right-handed healthy volunteers (4 men, 8 women, 57.4 ± 11.3 years old). Stroke patients were included if they met the following criteria: (a) had experienced a first-ever stroke at least 6 months prior to recruitment; (b) had an acute unilateral loss of right-hand grip strength score of <4 on the Medical Research Council scale (0–5, with 5 being normal) for at least 48 h; and (c) were right-handed according to the Edinburgh Handedness Inventory. Exclusion criteria included: (a) any hearing, vision, language, or cognitive deficit; (b) contraindications for fMRI; and (c) any disorder that impairs motor function of the stroke-affected hand. Institutional review board approval for the study was granted by the Partners Human Research Committee (protocol no. 2005P000570), and all participants provided informed consent.

### 2.2 Rehabilitation protocol

Patients were supervised at home while receiving training with the third-generation Magnetic Resonance Compatible Hand-Induced RObotic Device (“MR_CHIROD”) (21), which was coupled with an interactive computer game as described in detail elsewhere (22). Each patient underwent training for 45 min per day, 3 days per week, over a period of 10 weeks. Each training session included four 8-min scenarios separated by short rest breaks. In the game, users control a small green alien in a flying saucer as it navigates through a scrolling labyrinth. The saucer’s speed gradually increases as the game progresses. Players earn points for avoiding obstacles and collecting rewards in the game. The saucer’s altitude is controlled by squeezing and releasing the hand-gripper part of the MR_CHIROD, with the handles’ position directly corresponding to the saucer’s height on the screen. The objective of the game is to maximize one’s score by avoiding obstacles and collecting rewards. Motor performance was evaluated before training (baseline), at approximately monthly intervals during training to track progress, and 1 month after completing training (follow-up) to assess persistence over time. To evaluate upper limb function, the Fugl-Meyer upper extremity (FM-UE) scale was used to assess sensorimotor impairment, while the Modified Ashworth scale was used to assess spasticity.

### 2.3 Imaging

The chronic stroke patients underwent brain scans before, during the rehabilitation training, and at the 1-month follow-up, in concurrence with clinical motor assessments. The PPI analysis was conducted on the baseline scans before rehabilitation. The healthy subjects, on the other hand, only underwent a single MRI scan. All brain imaging data were collected using a 3-T Skyra Siemens full-body scanner equipped with a 32-channel phased-array head coil. The imaging protocol included: (a) a high-resolution T1-weighted magnetization-prepared rapid gradient-echo sequence with a repetition time (TR) of 2,300 ms, an echo time (TE) of 2.53 ms, an inversion time of 900 ms, a field of view (FOV) of 256 mm, a resolution of 1 × 1 × 1 mm^3^, and a PAT factor of 2 for anatomical imaging; (b) a double-echo fast gradient echo pulse sequence (TR, 650 ms; TE1, 4.92 ms; TE2, 7.38 ms; FOV, 220 mm; resolution, 2 × 2 × 2 mm^3^) for field mapping; and (c) a single-shot multi-slice echo-planar imaging pulse sequence (TR, 3,000 ms; TE, 30 ms; FOV, 220 mm; resolution, 2 × 2 × 2 mm^3^; Parallel Acquisition Techniques (PAT) factor, 2; simultaneous multi-slice shift, 3; 100 dynamic scans; and 4 dummy scans) for fMRI.

### 2.4 Motor task

The motor task followed a classical boxcar design that alternated between 21-s rest and action periods. During the seven action periods, participants used their right hand to compress and continuously release the hand-gripper part of the robotic device at a rate of 0.52 Hz. The squeezing rate was guided by a visual ‘metronome’ cue circle, which oscillated radially at a frequency of 0.52 Hz (i.e., 11 squeezes per action period) and was projected onto a neutral-background screen. A fixation cross was displayed during the seven rest periods. To minimize motion and dampen motion coupling between the subject’s arm and body, foam rubber pads with straps across the forehead, arms, and elbows were used. Additionally, the non-moving hand was closely monitored for any mirror motions.

### 2.5 Data analysis

The data were processed and analyzed using the CONN toolbox (23). Preprocessing involved susceptibility distortion correction, motion correction, slice-timing correction, outlier identification, co-registration with the T1 weighted image, tissue-class segmentation, normalization to Montreal Neurological Institute (MNI) standard space and smoothing with an 8 × 8 × 8 mm^3^ Gaussian kernel. The anatomical component-based noise correction procedure (aCompCor) method and temporal band-pass filtering, both available in the CONN toolbox (24), were employed to denoise the data and remove physiological and movement components from the BOLD signal. The aCompCor method regressed out noise components from cerebral white matter and cerebrospinal areas, subject-motion parameters, which were estimated from the registration procedure, outlier scans or scrubbing, constant and first-order linear session effects and constant task effects. Additionally, temporal frequencies below 0.008 Hz or above 0.09 Hz were filtered out from the BOLD signal. This step aimed to focus on slow-frequency fluctuations while minimizing the influence of physiological, head-motion, and other noise sources.

To examine the level of motor-modulated effective connectivity between left or right VTA and every voxel in the brain, a seed-to-voxel PPI connectivity analysis was conducted. Right and left VTA were defined by thresholding at 50% a midbrain probabilistic atlas (25). A general linear model was used to describe the signal of every voxel in the brain using three terms: the motor task box-car function convolved with a canonical hemodynamic response function (psychological factor); (b) the average signal from the left or right VTA (physiological factor) and; (c) the interaction term specified as the product of (a) and (b) (PPI term).

At the group level, three mass univariate voxel-wise analyses were conducted. The first analysis compared PPI differences between healthy subjects and chronic stroke patients, while the other two analyses examined the associations between PPI and baseline FM-UE values or D(FM-UE) = (FM-UE)_follow-up_ – (FM-UE)_baseline_, i.e., differences between baseline and follow-up FM-UE values. Age and gender were included as covariates in all analyses. Cluster-based inferences were performed with false discovery rate parametric statistics for family-wise error control at *p* = 0.05.

## 3 Results

**Figure 1:**
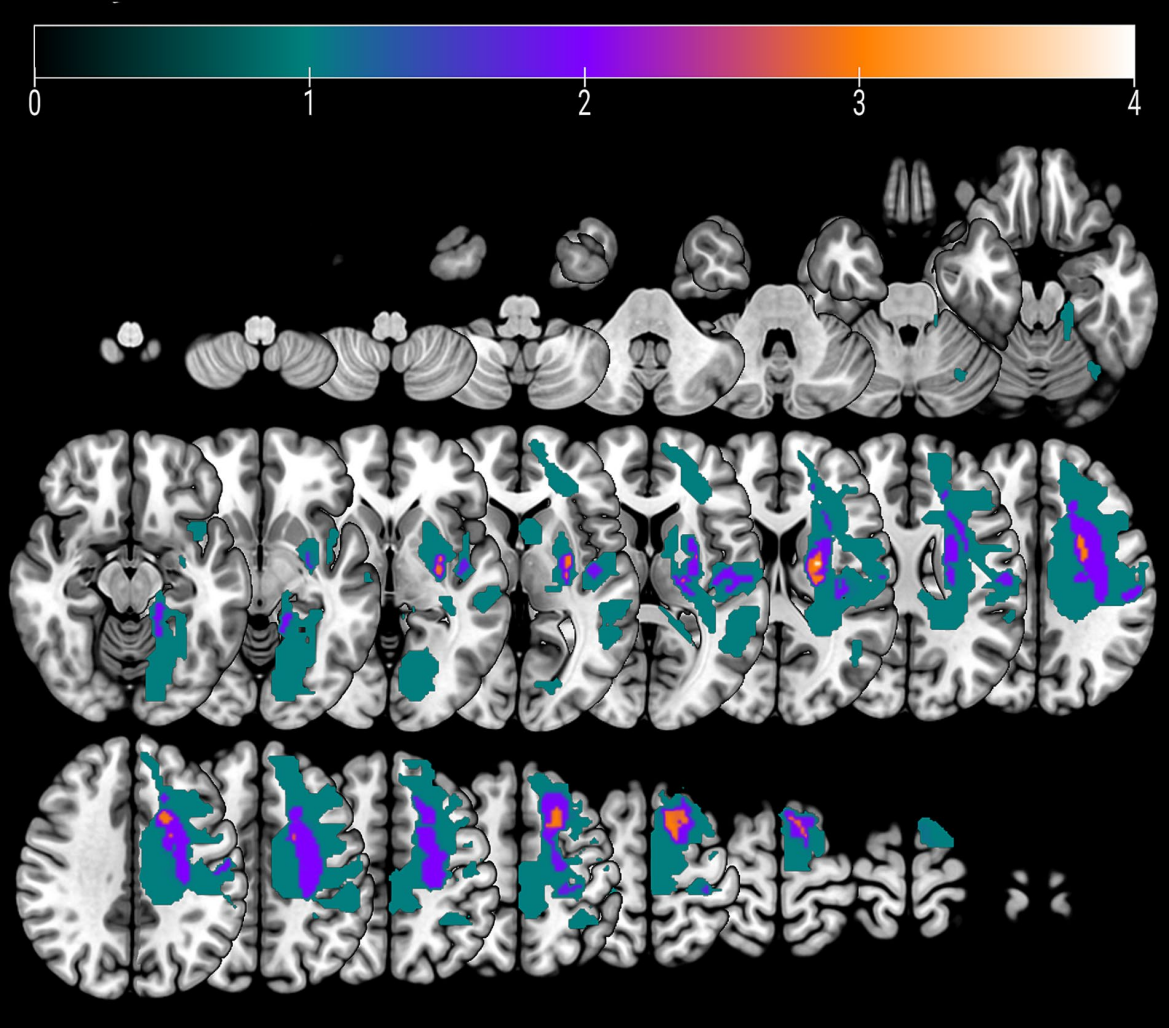
An overlay color map of individual stroke lesions of all 12 patients on a T1-template. Color scale indicates the number of patients having a lesion in this voxel.

Five patients experienced cortical strokes, while seven patients had subcortical strokes. Two of the strokes were hemorrhagic, while the remainder were ischemic. The overlay of the stroke lesions of all patients is shown in Figure 1. All patients successfully completed the rehabilitation program. Table 1 shows demographics and clinical data of all patients. FM-UE scores indicated that all patients exhibited clinically significant right-sided motor impairment, with a mean FM-UE score of 27.6 ± 8.7. One patient displayed spasticity. Four patients demonstrated improvement at the conclusion of rehabilitation, with FM-UE progress scores of +3, +4, +5, and + 10, respectively.

**Table 1:** Demographics and clinical data of chronic stroke patients.

No	Age (years)	Gender	Duration since stroke (months)	FM-UE	D(FM-UE)
1	39	F	19	27	+5
2	44	M	48	36	0
3	50	M	18	36	0
4	46	F	34	36	0
5	59	F	21	18	+3
6	61	F	114	15	+4
7	45	F	156	28	0
8	68	M	81	32	0
9	40	M	168	24	0
10	33	F	54	32	0
11	58	M	70	12	+10
12	35	F	28	36	0

F, Female; M, Male; FM-UE, Fugl-Meyer upper extremity scale; D(FM-UE), difference of FM-UE between follow-up and baseline.

**Figure 2:**
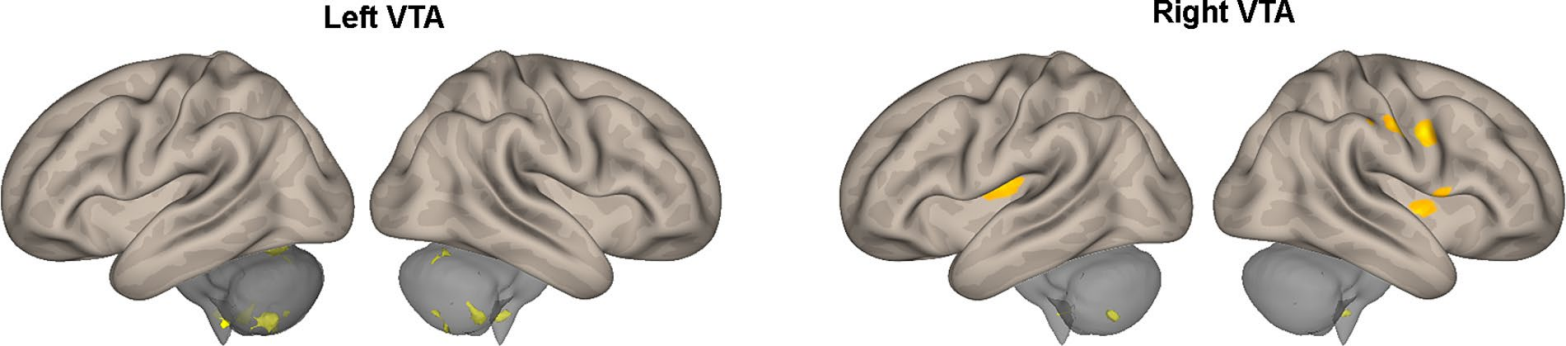
Areas of Table 2 exhibiting greater effective connectivity with the left or right VTA in healthy subjects compared to stroke patients. The areas, highlighted in color, are superimposed on the lateral views of a semi-inflated white matter surface of a brain atlas.

Table 2 provides a summary of the brain areas exhibiting significantly higher PPI with VTA in healthy subjects compared to stroke patients. The corresponding brain regions are visually depicted in Figure 2. The corresponding brain regions are visually depicted in Figure 2. The results reveal two consistent patterns observed across all study findings. The first pattern is hemispheric asymmetry, as demonstrated by cerebellar regions associated with the ipsilesional VTA and cortical regions of the somatosensory cortex and insula linked to the contralesional VTA. The second pattern involves the presence of transhemispheric connectivity, where the VTA of one hemisphere is related not only to brain areas in the same hemisphere but also to areas within the other hemisphere.

**Table 2:** Brain areas where the average PPI with right (contralesional) and left (ipsilesional) VTA, after controlling for the influence of age and sex, is larger in healthy subjects than in chronic stroke patients.

Seed Area	Cluster location	BA	MNI coordinates	Cluster size	P-FDR
VTA L	Cerebelum L		−12 −64 −20	245	0.000017
	Cerebelum L		−20 −68 −54	200	0.000060
	Cerebelum R		+04–42 −54	90	0.007382
	Cerebelum R		+32–52 −50	88	0.007382
	Cerebelum R		+10–76 −52	87	0.007382
VTA R	Premotor Cortex, Primary motor Cortex R	6, 4	+58–02 + 34	169	0.000351
	Insular Cortex L	13	−38 −08 + 14	109	0.004017
	Thalamus R		+10–26 + 10	79	0.015886
	Cerebelum L		−18 −66 −50	69	0.020663
	Insular, IFC pars opercularis R	13, 44	+40 + 00 + 00	67	0.020663
	Brain Stem		+00–40 −50	58	0.031724
	Postcentral Gyrus L		−44 −16 + 28	55	0.033566

BA, Brodmann Area; MNI, Montreal Neurological Institute; Assoc, Association; FDR, false discovery rate; R, right; L, left.

**Figure 3:**
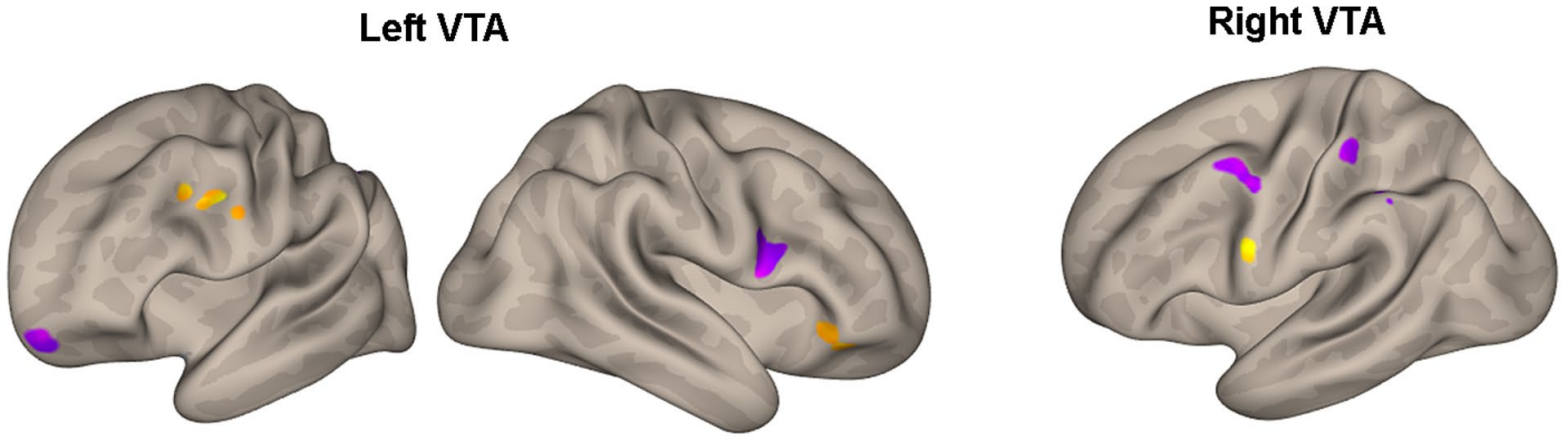
Areas of Table 3 with significant association between the effective connectivity with the left or right VTA and FM_UE score. The areas, highlighted in yellow for positive associations and in blue for negative associations, are overlaid on semi-inflated white matter hemispheric surfaces of a brain atlas.

Table 3 and Figure 3 present the brain areas where the PPI with VTA shows significant associations with the FM-UE score. The results reveal significant associations between motor performance and effective connectivity with both the right and left VTA. Cortical areas in the brain associated with motor functions (i.e., premotor and primary motor cortex) and reward processing (i.e., frontal and prefrontal cortex) exhibited notable relationships with VTA activity.

**Table 3:** Brain areas where the average PPI with right (contralesional) and left (ipsilesional) VTA, after controlling for the influence of age and sex, is association with FM-UE score.

Seed Area	Cluster location	BA	MNI coordinates	Cluster size	Assoc	P-FDR
VTA L	Inferior Frontal Gyrus, pars opercularis R	9, 13, 6	+50 + 06 + 08	64	−	0.000028
	Inferior Prefrontal Gyrus R	47	+36 + 32–08	53	+	0.000089
	Supramarginal Gyrus, Arcuate Anterior Segment L	47	−38 -34 + 26	30	+	0.004537
	Anterior Prefrontal Cortex L	10	−16 + 62–16	23	−	0.015675
	Premotor Cortex, Middle Frontal Gyrus L	10	−36 + 10 + 32	22	+	0.015785
	Dorsolateral Prefrontal Cortex, Premotor Cortex L	9, 6	−62 + 10 + 28	19	+	0.026820
	Supramarginal Gyrus L	40	−52 −46 + 52	18	−	0.029390
VTA R	Premotor Cortex, Supramarginal Gyrus, Dorsolateral Prefrontal Cortex L	6, 4	−52 −34 + 56	69	−	0.000002
	Premotor Cortex Primary Motor Cortex L	6, 4	−48 + 06 + 54	60	−	0.000005
	Premotor Cortex Dorsolateral Prefrontal Cortex L	6, 9	−60 + 10 + 30	27	+	0.002850

BA, Brodmann Area; MNI, Montreal Neurological Institute; Assoc, Association; FDR, false discovery rate; R, right; L, left.

**Figure 4:**
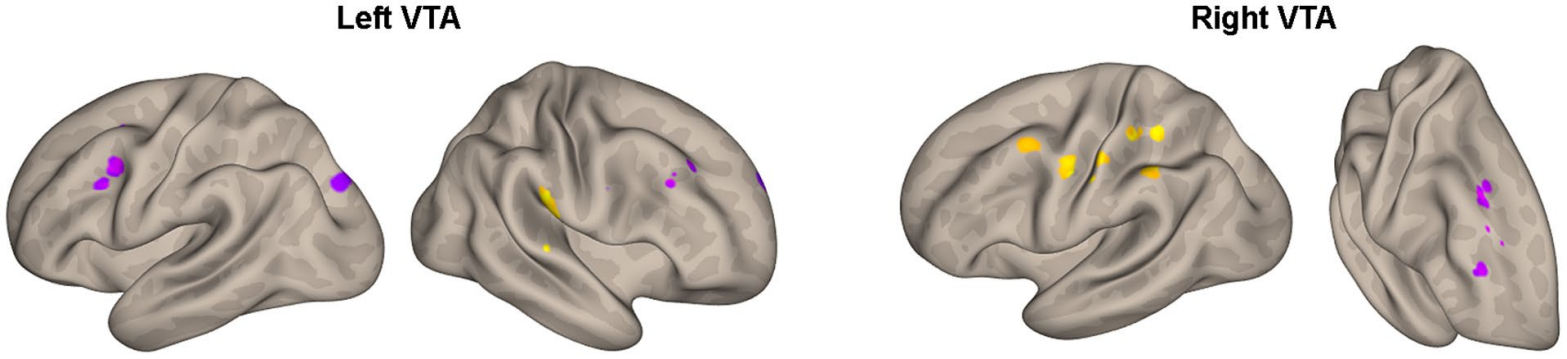
Areas of Table 4 with significant association between the effective connectivity with the left or right VTA and changes in FM_UE score. The areas, highlighted in yellow for positive associations and in blue for negative associations, are overlaid on semi-inflated white matter hemispheric surfaces of a brain atlas.

Furthermore, Table 4 and Figure 4 reveal the brain areas in which the PPI with VTA is significantly associated with D(FM-UE), representing the change in FM-UE score over time. The findings reveal an asymmetrical pattern: positive associations between PPI and FM-UE changes are observed between areas in one hemisphere and the opposite-side VTA, while negative associations occur with the VTA on the same side. Areas associated with changes in FM-UE belong to the same cortices (motor, premotor, frontal, and prefrontal) as areas related to FM-UE.

**Table 4:** Brain areas where the average PPI with right (contralesional) and left (ipsilesional) VTA, after controlling for the influence of age and sex, is associated with FM-UE changes D(FM-UE).

Seed Area	Cluster location	BA	MNI coordinates	Cluster size	Assoc	P-FDR
VTA L	Premotor Cortex L	6	−32 + 08 + 42	109	−	0.000000
	Associative Visual Cortex, Secondary Visual Cortex L	19, 18	−18 −86 + 36	41	−	0.000865
	Insular Cortex, Supramarginal Gyrus R	13, 40	+56–18 + 18	36	+	0.001510
	Dorsal Frontal Cortex R	8	+38 + 38 + 38	25	−	0.011210
	Dorsal Frontal Cortex, Dorsolateral Prefrontal Cortex R	8, 9	+52 + 22 + 36	23	−	0.014071
	Dorsolateral Prefrontal Cortex R	9	+10 + 64 + 28	19	−	0.030090
VTA R	Supramarginal Gyrus, Premotor Cortex L	40	−40 −38 + 52	110	+	0.000000
	Primary Somatosensory Cortex, Primary Motor Cortex L	2, 3, 4	−52 −10 + 44	38	+	0.000275
	Dorsal Frontal Cortex R	8	+26 + 26 + 42	29	−	0.001317
	Premotor Cortex L	6	−38 + 08 + 58	28	+	0.001317
	Dorsolateral Prefrontal Cortex, Dorsal Frontal Cortex R	9,8	+34 + 44 + 30	22	−	0.004657

BA, Brodmann Area; MNI, Montreal Neurological Institute; Assoc, Association; FDR, false discovery rate; R, right; L, left.

## 4 Discussion

During the application of PPI analysis in a rehabilitative motor task, there was detected motor modulation of the effective connectivity between the ventral tegmental area and several brain regions. The extent of modulation varied between individuals with CSPs and HCs. In CSPs, the degree of modulation was associated with the motor performance index and could predict the impact of rehabilitation on their motor performance.

Contrary to most PPI studies that restrict their analysis to task-activated areas, we have chosen the VTA as a seed region, despite it not being activated during the motor task in any of our subjects. This choice is supported by the understanding, as explained by Gershen et al. (26), that the relationship between activation and connectivity of a brain area is not straightforward, allowing for independent changes in each of these aspects. In our study, we have provided evidence to support this assertion by demonstrating motor-related connectivity changes among non-activated regions. This finding highlights the occurrence of broader dynamic reconfiguration of a large-scale reward-related brain network during a simple motor task, which can be detected through PPI analysis.

After analyzing the left and right VTA separately, it was found that VTA influence was asymmetric in all the results, with the unaffected contralateral hemispheric connections playing an active role. Asymmetries in the dopaminergic system of healthy humans have been associated with lateralized motor performance and hand preference (27–29). Previous animal studies have demonstrated that stroke induces changes in the dopamine circuit. A study reported the loss of ipsilesional dopaminergic neurons within the VTA after a photothrombotic stroke within M1, indicating that these changes may occur even if the lesions are far from the VTA, through a mechanism called exofocal dopaminergic degeneration (30). These changes might trigger compensatory/regulatory mechanisms in the contralesional hemisphere (31). A previous study with mice indicated that the unilateral injection of pharmacological agents decreasing dopaminergic transmission of the VTA results in depression of cortical activity in the prefrontal cortex, mainly in the hemisphere contralateral to the VTA (31). Many studies applying electrical or pharmacological stimulation of the unaffected VTA (also known as contralateral facilitation) have shown the restoration of VTA-dependent behaviors like feeding or exploratory locomotion (32). Consistent with these studies, the comparison between groups showed that the contralesional VTA appears disassociated with more regions in CSPs than HCs, including those related to motor function, in both hemispheres. This finding supports the idea of a whole-brain dopaminergic degeneration in chronic stroke with an important role of the contralesional VTA. The disassociated areas are either directly connected to the VTA (thalamus, brain stem, insular cortex) or receive inputs from other regions involved in reward processing, such as the prefrontal cortex (premotor cortex, primary motor cortex, post central gyrus) (Table 2). The absence of findings in the ipsilesional hemisphere could be explained by the spatial variability of the stroke lesions which does not allow group-level statistical convergence at particular locations.

The between-group comparison also revealed that there was a stronger PPI between the ipsilesional VTA and cerebellum in HCs compared to CSPs (Table 2; Figure 2). Recent studies have suggested that these two regions have bidirectional connections that may facilitate their interaction in motor learning and control (33, 34). These findings highlight the potential importance of VTA-cerebellum interactions in motor function and suggest that disruptions in these connections in the affected hemisphere may be relevant to the motor impairments experienced by CSPs.

In the CSPs’ group, motor performance was found to be associated with the motor-related modulation of the ventral tegmental area (VTA) in various areas related to the motor task and the reward process (Table 3; Figure 3). Notably, the higher FM-UE score in stroke patients’ performance is associated with a more pronounced influence of both the right and left VTA on areas in the premotor and dorsolateral prefrontal cortex of the ipsilesional hemisphere. The motor cortices (primary motor and premotor) are primarily involved in planning and executing movements but have also been found to encode reward-related feedback (35, 36). The dorsolateral prefrontal cortex is responsible for executive functions, working memory, cognitive control, and decision-making. It possesses reciprocal connections with the VTA and other mesolimbic dopaminergic regions, enabling the integration of cognitive control processes with reward-related information (37). Similar to the premotor cortex, the connectivity detected between the dorsolateral prefrontal cortex and the VTA appears to be stronger in patients with better motor performance. The same holds true for the inferior prefrontal cortex and the supramarginal gyrus. In general, the orbitofrontal cortex is implicated in learning, prediction, and decision-making for emotional and reward-related behaviors (38). Specifically, the inferior prefrontal cortex (BA 47) contributes to processing changes in reward-related information (39).

On the other hand, motor performance was negatively associated with the PPI between the ipsilesional VTA and the contralesional inferior frontal gyrus, an area known to be related to the ability to inhibit an already-initiated action (i.e., inhibitory control) (40). This relationship might reflect the inverse association between motor performance and the number of potential errors during the execution of the motor task, as well as the increased self-reward experienced by more disabled subjects when they successfully correct these errors. Negative associations between motor performance and the interactions of the contralesional VTA with the motor cortices (BA4, 6) of the contralesional hemisphere indicate the enhanced and potentially compensatory role of the contralesional hemisphere in the motor-related reward processes of the more disabled patients.

Notably, the influence of the VTA on the motor circuitry is associated with changes in FM-UE scores, which quantify motor performance improvement resulting from rehabilitation (Table 4; Figure 4). Again, an asymmetric pattern emerges: positive associations between PPI and FM-UE changes are observed between areas in one hemisphere and the contralateral VTA, while negative associations are observed with the ipsilateral VTA (Table 4). Specifically, regions within the motor circuitry of the affected hemisphere, such as the motor and premotor cortex, exhibit stronger modulation by the contralesional VTA when there is a higher increase in FM-UE scores. Conversely, the ipsilesional VTA demonstrates a stronger influence on the ipsilesional premotor cortex in patients with poorer rehabilitation outcomes. The same cross-hemispheric dependence of the motor-related areas with the VTA in the case of successful rehabilitation appears for brain regions in the dorsal frontal and dorsolateral prefrontal cortex. In other words, activation of the self-reward system in the ipsilesional hemisphere, originating from the contralesional VTA, contributes to improved rehabilitation outcomes. Previous research has linked external rewards with motor learning and rehabilitation (4). However, this study demonstrates the importance of self-reward processes in rehabilitation as well. Importantly, it shows that utilizing neuroimaging techniques allows for the quantification of these processes and their potential to predict rehabilitation outcomes. A natural extension of the current study would be to integrate neuroimaging with psychometric trackers to quantify the intrinsic motivations of subjects. This integration can deepen our understanding of the role of motivation and reward systems in stroke recovery and pave the way for more effective and personalized rehabilitation approaches.

This study has several limitations that should be acknowledged. The sample size was relatively small. However, this was designed and should be considered as an exploratory study on the ability of PPI analysis to assess VTA connectivity using fMRI data of a simple motor task. Given the small sample size, the results should be replicated in larger cohorts before generalization. Also, there is considerable variability in stroke lesions, which may lead to inconsistent perturbation of the VTA connectivity network. The heterogeneity of stroke lesions can complicate the interpretation and generalizability of the results. The PPI analysis employed in this study does not account for the influences of other important areas within the reward network, such as the substantia nigra. Additionally, the PPI cannot establish causal relationships between the VTA and other areas, particularly when there is bidirectional connectivity, as is the case with the prefrontal cortex. As a result, it remains unclear whether frontal and prefrontal areas regulate the release of dopamine from the VTA and modulate reward processing, or if dopamine release from the VTA impacts their functioning, influencing cognitive processes, motivation, and attention. Despite these limitations, our results show that PPI could be a valuable tool in elucidating the functional interactions and network dynamics within the brain, yielding important insights into how different brain regions interact and modulate their connectivity in response to simple rehabilitation exercises.

In conclusion, the assessment of self-reward processes during rehabilitation of chronic stroke patients can be accomplished through PPI analysis of the VTA brain connectivity. Various regions within the motor circuitry, cerebellum, and prefrontal cortex are implicated in these processes, with the contralesional hemisphere assuming a greater role as motor performance declines. Additionally, the effective connectivity of the VTA holds promise as a potential predictor of motor recovery, highlighting the significance of psychological factors in the rehabilitation process.

## Data availability statement

The raw data supporting the conclusions of this article will be made available by the authors, without undue reservation.

## Ethics statement

The studies involving humans were approved by Partners Human Research Committee (protocol no. 2005P000570). The studies were conducted in accordance with the local legislation and institutional requirements. The participants provided their written informed consent to participate in this study.

## Author contributions

LA: Conceptualization, Data curation, Formal analysis, Investigation, Methodology, Software, Validation, Visualization, Writing – original draft. SE: Data curation, Investigation, Methodology, Resources, Writing – review & editing. IG: Investigation, Methodology, Validation, Visualization, Writing – review & editing. SL: Data curation, Investigation, Resources, Validation, Visualization, Writing – review & editing. HB: Formal analysis, Investigation, Validation, Visualization, Writing – review & editing. AT: Investigation, Validation, Visualization, Writing – review & editing, Conceptualization, Funding acquisition, Methodology, Project administration, Supervision.
